# Characteristics of NtCCD1-3 from tobacco, and protein engineering of the CCD1 to enhance ***β***-ionone production in yeast

**DOI:** 10.3389/fmicb.2022.1011297

**Published:** 2022-09-23

**Authors:** Xiaowei Gong, Fan Li, Yupeng Liang, Xiulin Han, Mengliang Wen

**Affiliations:** ^1^National Key Laboratory for Conservation and Utilization of Bio-Resources in Yunnan, Key Laboratory of Microbial Diversity in Southwest China, Ministry of Education, School of Life Sciences, Yunnan Institute of Microbiology, Yunnan University, Kunming, China; ^2^R&D Center, China Tobacco Yunnan Industrial Co., Ltd., Kunming, China

**Keywords:** carotenoid cleavage dioxygenase 1, *Nicotiana tabacum*, ***β***-ionone, protein engineering, *Saccharomyces cerevisiae*

## Abstract

Biosynthesis of *β*-ionone by microbial cell factories has become a promising way to obtain natural *β*-ionone. The catalytic activity of carotenoid cleavage dioxygenase 1 (CCD1) in cleavage of *β*-carotene to *β*-ionone severely limits its biosynthesis. In this study, NtCCD1-3 from *Nicotiana tabacum* with high ability to cleave *β*-carotene was screened. Multiple strategies for improving the *β*-ionone yield in *Saccharomyces cerevisiae* were performed. The results showed that NtCCD1-3 could cleave a variety of caroteniods at the 9,10 (9′,10′) double bonds and lycopene at the 5,6 (5′,6′) positions. The insertion site delta for *NtCCD1-3* gene was more suitable for enhancing the yield of *β*-ionone, showing 19.1-fold increase compared with the rox1 site. More importantly, mutant K38A of NtCCD1-3 in membrane-bonding domains could greatly promote *β*-ionone production by more than 3-fold. We also found that overexpression of the NADH kinase Pos5 could improve *β*-ionone yield up to 1.5 times. These results may provide valuable references for biosynthesis of *β*-ionone.

## Introduction

*β*-Ionone is a kind of apocarotenoid that has violet-like, as well as mixed fruit and woody odor characteristics, with an extremely low aroma threshold of 0.007 nl/l in water (Baldwin et al., 2000), and 0.12 ng/l in air (Brenna et al., 2002). The molecular formula of *β*-ionone is C_13_H_20_O and the molecular mass is 192. *β*-Ionone widely exists in raspberries, tomatoes, petunias, melons, osmanthus, grapes and lychees (Simkin et al., 2004a,b; Ibdah et al., 2006; Mahattanatawee et al., 2007; Beekwilder et al., 2008; Baldermann et al., 2010; Ristic et al., 2010). The compound is used extensively in food, personal care products (Lalko et al., 2007), and the synthesis of *β*-carotene and vitamin A (Cataldo et al., 2016). In addition, *β*-ionone can inhibit the proliferation of breast cancer and gastric cancer cells (Duncan et al., 2004; Liu et al., 2013). According to statistics, from 2011 to 2015, the global average annual sales volume of *β*-ionone reached 166 million dollars and was still growing rapidly (Czajka et al., 2018).

At present, chemical synthesis is still the main way to obtain *β*-ionone, while people prefer natural *β*-ionone as it is safer and friendlier to environment. Natural *β*-ionone is mainly extracted from plants. However, the content of *β*-ionone in plants is relatively low, even in plants with higher concentrations, such as 19.8–31.9 μg/Kg in grapes and only 1.72 mg/Kg in raspberry (Beekwilder et al., 2008; Ristic et al., 2010). As a result, the yield of natural *β-*ionone is severely limited, and the price of natural *β*-ionone is 10–100 times higher than that of the chemically synthesized *β*-ionone (Nacke et al., 2012). Furthermore, due to uncontrollable natural conditions, the yield of *β*-ionone from plant extraction fluctuates greatly, which is difficult to meet the continuous and stable commercial demand. With the rapid development of biotechnology, it is possible to prepare *β*-ionone *via* the biosynthesis approach, which has become a research hotspot. Microorganisms (e.g., *Escherichia coli*, *Saccharomyces cerevisiae* and *Yarrowia lipolytica*) are usually employed as the host by using the mevalonate (MVA) pathway for *β*-ionone biosynthesis. The pathway of *β*-ionone biosynthesis in engineered microorganisms is shown in Supplementary Figure 1.

Zhang et al. (2018) reconstructed the pathways of MVA and *β*-carotene synthesis in *E. coli* BL21-Gold DE3. Through heterologous expression of PhCCD1 from *Petunia hybrida* and metabolic engineering optimization, the *β*-ionone titer of the engineered strain reached 500 mg/l in the fed-batch fermentation. Beekwilder et al. (2014) expressed the *β*-carotene synthesis genes (*crtE*, *crtYB* and *crtI*) from *Xanthophyllomyces dendrorhous* and *RiCCD1* gene from raspberry in *S. cerevisiae* cen. pk, and the yield of *β*-ionone was 0.22 mg/g DCW. López et al. (2015) overexpressed truncated hydroxymethylglutaryl-CoA reductase (tHMGR), geranylgeranyl phosphate synthase (BST1), phytoene synthase/lycopene cyclase (crtYB), phytoene dehydrogenase (crtI) and PhCCD1 using *S. cerevisiae* SCIGS22 as the chassis, and the yield of *β*-ionone was 0.63 mg/g DCW in shake flask, and 1 mg/g DCW in the fed-batch fermentation. Similarly, Naseri et al. (2019) expressed *BTS1*, *crtYB*, *crtI*, and *RiCCD1* genes in *S. cerevisiae* MXFde 0.2 by means of COMbinatorial Pathway ASSembly (COMPASS), and the highest yield of *β*-ionone approached 0.2 mg/g DCW. Based on the oleaginous *Y. lipolytica* CLIB138, Czajka et al. (2018) overexpressed a series of genes in the MVA pathway, and heterologously expressed phytoene dehydrogenase (*carB*) and lycopene cyclase/phytoene synthase (*carRP*) genes from *Mucor circinelloides* and *OfCCD1* gene from *Osmanthus fragrans*. Finally, the *β*-ionone titer of the engineered yeast was 68 mg/l in shake flask, and 380 mg/l in the fed-batch fermentation. Lu et al. (2020) also expressed above *carB*, *carRP* and *PhCCD1* genes in *Y. lipolytica* Po1f chassis through modular pathway engineering strategy, resulting in a titer of *β*-ionone 358 mg/l in the shake-flask fermentation and 980 mg/l in the fed-batch fermentation, which is the highest yield of *β*-ionone to date. However, the yield of *β*-ionone synthesized by microorganisms has not reached the gram level, which is still difficult to meet the demand of industrial production. Therefore, it is particularly urgent to find the key gene elements that can improve the yield of *β*-ionone. Moreover, previous paper has suggested that the CCDs catalyzed reaction was the limiting step for *β*-ionone biosynthesis (Czajka et al., 2018). However, the research on high activity CCDs from plant is also insufficient, so it is very important to explore other CCDs with high activity to cleave *β*-carotene to produce *β*-ionone.

Since a protein’s structure determines its activity, many structures of CCDs have also been resolved to understand the molecular mechanism behind them. The CCD proteins all share a seven bladed *β*-propeller structure with ferrous ion in its center. As a cofactor, the ferrous ion is coordinated bonding with four conserved histidine residues to form its active site (Messing et al., 2010). The *α*1 and *α*3 helices are mainly composed of hydrophobic amino acids and are the membrane-binding regions of CCD proteins (Tan et al., 2001). The two helices form the domes of CCD proteins. The precursors of *β*-ionone, such as *β*-carotene, are all hydrophobic compounds and mostly exist in the membrane structures of cells. Therefore, if the membrane binding ability of CCDs is improved, the binding probability between CCDs and substrates may be increased, which may be beneficial to increase the yield of the products. This has been proved to be reasonable by relevant studies. For example, when a glycerol-conducting channel protein was fused with the membrane-binding domain of PhCCD1 and expressed in *E. coli*, the yield of *β*-ionone was 2 mg/g DCW, which was nearly 4-fold than that of the unmodified protein (Ye et al., 2018). Similarly, Werner et al. (2019) obtained chimeric proteins lck-PhCCD1, fyn-PhCCD1, and mutant protein K164L by protein engineering modification of the membrane-binding domain of PhCCD1. After expression of these modified proteins in *S. cerevisiae*, the titer of *β*-ionone was at least 3-fold higher than that of the native PhCCD1. Whereas, the deletion of the first 43 residues (part of membrane-binding domain) at the N-terminus of PhCCD1 led to no *β*-ionone detected (Werner et al., 2019). These studies indicated that improving the membrane binding ability of CCDs to increase their activity is an effective way to increase the yield of *β*-ionone. However, there are few studies on improving the membrane binding ability of CCDs by site-directed protein mutation for increasing the yield of *β*-ionone.

Tobacco (*Nicotiana tabacum*) is an important industrial crop and a model species for biological studies. As an aromatic plant, tobacco contains high levels of carotenoid degradation products in its essential oil, in which *β*-ionone is up to 1.08 mg/100 g (Popova et al., 2019). The reason may be that CCDs proteins with high catalytic activity exist in the tobacco. Regrettably, there are few public reports involving catalytic activity of NtCCDs from tobacco. In this study, a tobacco CCD1 (*NtCCD1-3*) with high ability to cleave *β*-carotene was selected and characterized. To biosynthesize *β*-ionone by using NtCCD1-3, the homology model of NtCCD1-3 structure was constructed, and some key amino acid residues at membrane-binding regions were selected for site-directed mutagenesis to investigate their relationship with the activity. This study may enhance the insight of CCD1 proteins and provide valuable references for the biosynthesis of *β*-ionone and other valuable apocarotenoids in yeast.

## Materials and methods

### Strains, plasmids, and chemicals

All strains and plasmids used in this study are shown in Table 1. *β*-Ionone (>97%) was purchased from Sigma-Aldrich. pEASY^®^-Basic Seamless Cloning and Assembly Kit and PrimeScript™ RT reagent Kit were purchased from TaKaRa. TIANgel Midi Purification Kit was purchased from TianGen Biotech Co., Ltd. (Beijing, China). E.Z.N.A.^®^ Plant RNA Kit and 2 × TSINGKE® Master qPCR Mix (SYBR Green I) Kit were purchased from Omega Bio-Tek and Tsingke Biotechnology Co., Ltd. (Beijing, China), respectively. Dodecane (analytical standard) and other reagents were purchased from aladdin (Shanghai, China).

**Table 1:** Strains and plasmids used in this study.

Strains/plasmids	Description	Source
**Strains**		
BY4741	MATα his3Δ1 leu2Δ0 met15Δ0 ura3Δ0	Yuchun Biology
A10	BY4741 *ura3*::P_GAP_-crtE-T_CYC1_,P_GAP_-crtYB-T_CYC1_,P_GAP_-crtI-T_CYC1_	This study
A10-rox1-NtCCD1-3	A10 *rox1*::P_ADH1_-NtCCD1-3-T_CYC1_, P_Leu2_-Leu2-T_Leu2_	This study
A10-rox1-PhCCD1	A10 *rox1*::P_ADH1_-PhCCD1-T_CYC1_, P_Leu2_-Leu2-T_Leu2_	This study
A10-*δ*-NtCCD1-3	A10 *δ*::P_ADH1_-NtCCD1-T_CYC1_, P_Leu2_-Leu2-T_Leu2_	This study
A10-rox1-K25A	A10 *rox1*::P_ADH1_-K25A-T_CYC1_, P_Leu2_-Leu2-T_Leu2_	This study
A10-rox1-K31A	A10 *rox1*::P_ADH1_-K31A-T_CYC1_, P_Leu2_-Leu2-T_Leu2_	This study
A10-rox1-K38A	A10 *rox1*::P_ADH1_-K38A-T_CYC1_, P_Leu2_-Leu2-T_Leu2_	This study
A10-rox1-K42A	A10 *rox1*::P_ADH1_-K42A-T_CYC1_, P_Leu2_-Leu2-T_Leu2_	This study
A10-rox1-S128A	A10 *rox1*::P_ADH1_-S128A-T_CYC1_, P_Leu2_-Leu2-T_Leu2_	This study
A10-rox1-K140M	A10 *rox1*::P_ADH1_-K140M-T_CYC1_, P_Leu2_-Leu2-T_Leu2_	This study
A10-rox1-T155M	A10 *rox1*::P_ADH1_-T155M-T_CYC1_, P_Leu2_-Leu2-T_Leu2_	This study
A10-rox1-Y157A	A10 *rox1*::P_ADH1_-Y157A-T_CYC1_, P_Leu2_-Leu2-T_Leu2_	This study
A10-rox1-Y157L	A10 *rox1*::P_ADH1_-Y157L-T_CYC1_, P_Leu2_-Leu2-T_Leu2_	This study
A10-rox1-K164L	A10 *rox1*::P_ADH1_-K164L-T_CYC1_, P_Leu2_-Leu2-T_Leu2_	This study
A10-rox1-NtCCD1-3-Pos5	A10 *rox1*::P_ADH1_-NtCCD1-3-T_CYC1_, P_Leu2_-Leu2-T_Leu2_, P_TEF1_-Leu2-T_ADH1_	This study
A10-rox1-NtCCD1-3-Uli1	A10 *rox1*::P_ADH1_-NtCCD1-3-T_CYC1_, P_Leu2_-Leu2-T_Leu2_, P_GAP_-Leu2-T_CYC1_	This study
**Plasmids**		
tpLADH1	ColE1 origin, F1 origin, P_ADH1_, P_LEU_-LEU2-T_LEU_, Amp^R^	Wang et al. (2021)
tpLADH1-NtCCD1-3	tpLADH1 carrying P_ADH1_-NtCCD1-3-T_CYC1_, P_LEU_-LEU2-T_LEU_	This study
tpLADH1-PhCCD1	tpLADH1 carrying P_ADH1_-PhCCD1-T_CYC1_, P_LEU_-LEU2-T_LEU_	This study
T1	Cloning vector, pUC origin, *lac* Z, Amp^R^, Kan^R^	TransGen Biotech
T1-rox1	T1 Cloning vector carrying *rox1* from *S. cerevisiae*	This study
pColdTF	ColE1 origin, His-Tag, *lac* I, Amp^R^	TaKaRa
pColdTF-rox1-NtCCD1-3-LEU2	Derived from pColdTF, contains Leu and NtCCD1-3 cassette, rox1 homologous	This study
pColdTF-rox1-PhCCD1-LEU2	Derived from pColdTF, contains Leu and PhCCD1 cassette, rox1 homologous	This study
pColdTF-*δ*-NtCCD1-3-LEU2	Derived from pColdTF, contains Leu and NtCCD1-3 cassette, delta homologous	This study
T1-K25A	T1 cloning vector with the mutation K25A on full-length NtCCD1-3	This study
T1-K31A	T1 cloning vector with the mutation K31A on full-length NtCCD1-3	This study
T1-K38A	T1 cloning vector with the mutation K38A on full-length NtCCD1-3	This study
T1-K42A	T1 cloning vector with the mutation K42A on full-length NtCCD1-3	This study
T1-S128A	T1 cloning vector with the mutation S128A on full-length NtCCD1-3	This study
T1-K140M	T1 cloning vector with the mutation K140M on full-length NtCCD1-3	This study
T1-T155M	T1 cloning vector with the mutation T155M on full-length NtCCD1-3	This study
T1-Y157A	T1 cloning vector with the mutation Y157A on full-length NtCCD1-3	This study
T1-Y157L	T1 cloning vector with the mutation Y157L on full-length NtCCD1-3	This study
T1-K164L	T1 cloning vector with the mutation K164L on full-length NtCCD1-3	This study
pColdTF-rox1-K25A-LEU2	Derived from pColdTF-rox1-NtCCD1-3-LEU2, with the mutation K25A fragment replacing NtCCD1-3	This study
pColdTF-rox1-K31A-LEU2	Derived from pColdTF-rox1-NtCCD1-3-LEU2, with the mutation K31A fragment replacing NtCCD1-3	This study
pColdTF-rox1-K38A-LEU2	Derived from pColdTF-rox1-NtCCD1-3-LEU2, with the mutation K38A fragment replacing NtCCD1-3	This study
pColdTF-rox1-K42A-LEU2	Derived from pColdTF-rox1-NtCCD1-3-LEU2, with the mutation K42A fragment replacing NtCCD1-3	This study
pColdTF-rox1-S128A-LEU2	Derived from pColdTF-rox1-NtCCD1-3-LEU2, with the mutation S128A fragment replacing NtCCD1-3	This study
pColdTF-rox1-K140M-LEU2	Derived from pColdTF-rox1-NtCCD1-3-LEU2, with the mutation K140M fragment replacing NtCCD1-3	This study
pColdTF-rox1-T155M-LEU2	Derived from pColdTF-rox1-NtCCD1-3-LEU2, with the mutation T155M fragment replacing NtCCD1-3	This study
pColdTF-rox1-Y157A-LEU2	Derived from pColdTF-rox1-NtCCD1-3-LEU2, with the mutation Y157A fragment replacing NtCCD1-3	This study
pColdTF-rox1-Y157L-LEU2	Derived from pColdTF-rox1-NtCCD1-3-LEU2, with the mutation Y157L fragment replacing NtCCD1-3	This study
pColdTF-rox1-K164L-LEU2	Derived from pColdTF-rox1-NtCCD1-3-LEU2, with the mutation K164L fragment replacing NtCCD1-3	This study
pUC57-Uli1	pUC57 carrying P_GAP_-Uli1-T_CYC1_	Gifted by Yiyong Luo
tpLADH1-pos5	tpLADH1 carrying P_TEF1_-Pos5-T_ADH1_, P_LEU_-LEU2-T_LEU_	This study
pColdTF-Pos5-rox1	Derived from pColdTF, contains Leu, NtCCD1-3 and Pos5 cassette, rox1 homologous	This study
pColdTF-Uli1-rox1	Derived from pColdTF, contains Leu, NtCCD1-3 and Uli1 cassette, rox1 homologous	This study

### Phylogenetic analysis

NtCCD1-2 (AHH25650) and NtCCD1-3 (NP_001312918) from *N. tabacum* were selected as the candidate proteins for *β*-ionone biosynthesis. And PhCCD1 (AAT68189), AtCCD1 (CAA06712), ZmCCD1 (ABF85668), OfCCD1 (BAJ05401), VvCCD1 (AAX48772), RdCCD1 (ABY47994), CsCCD2 (AIG94929), CangCCD2 (ALM23546), CsCCD4 (ACD62476), AtCCD4 (AAM97019), CmCCD4 (ABY60885), ZmCCD7 (NP_001183928), AtCCD7 (AEC10494), CsCCD8a (AIF27229), AtCCD8 (NP_195007), VP14 (O24592), AtNCED3 (NP_188062), AtNCED5 (NP_174302), AtNCED6 (NP_189064) and AtNCED9 (NP_177960) distributed in CCD1, CCD2, CCD4, CCD7, CCD8, NCED3, NCED5, NCED6 and NCED9 respectively, were selected for constructing a phylogenetic tree by using MmRPE65 (AAL01119) as outgroup. All the amino acids sequences of the proteins above were downloaded from the NCBI protein database. Multiple protein sequences alignments were performed by using CLUSTAL X (Thompson et al., 1997). Neighbor joining phylogenetic tree using 1,000 bootstrap replications was generated in MEGA X, which was based on the multiple alignments of protein sequences above (Kumar et al., 2018).

### Expression of NtCCD1-3 and PhCCD1 in ***β***-carotene producing yeast

Previously, a *β*-carotene producing strain A10, in which the *crtE*, *crtYB*, and *crtI* genes from *X. dendrorhous* were expressed, was constructed by using *S. cerevisiae* BY4741 as the start strain. *NtCCD1-3* and *PhCCD1* genes were codon-optimized for *S. cerevisiae* expression. These genes were synthesized by GenScript (Nanjing, China), and cloned into the vector tpLADH1 to acquire tpLADH1-NtCCD1-3 and tpLADH1-PhCCD1, respectively. The integration plasmids of NtCCD1-3 and PhCCD1 at *rox1* locus were constructed and assembled, respectively, in *S. cerevisiae* according to the method described previously (Wang et al., 2021). First, the full-length *Rox1* gene was amplified from genomic DNA of *S. cerevisiae* BY4741 by using the primers rox1-F and rox1-R. Then, the *Rox1* gene was cloned into the vector T1 generating plasmid T1-rox1. The upstream 500 bp of *Rox1* was amplified from plasmid T1-rox1 by using primers rox1up-F and rox1up-R, and the 607 bp downstream arm of *Rox1* was amplified by using primers rox1down-F and rox1down-R. NtCCD1-3 expression cassette containing promoter *ADH1*, *NtCCD1-3* gene and *CYC1* terminator was obtained by amplification from plasmid tpLADH1-NtCCD1-3 using primers NtCCD1-3-infusion-F and NtCCD1-3-infusion-R. Primers LEU-infusion-F and LEU-infusion-R were used to amplify the LEU2 cassette as a selectable marker from plasmid tpLADH1-NtCCD1-3. Then *Eco*R I linearized vector pColdTF and the five fragments mentioned above were infused by using pEASY®-Basic Seamless Cloning and Assembly Kit to generate the expression vector pColdTF-rox1-NtCCD1-3-LEU2. The transformants were screened on LB agar plate containing 50 μg/ml ampicillin. The recombinant plasmid pColdTF-rox1-NtCCD1-3-LEU2 was extracted and confirmed by *Sma* I and *Pst* I digestion. After digestion with *Sma* I and *Pst* I, the rox1-up-P_ADH1_-NtCCD1-3-T_CYC1_-P_LEU_-LEU2-T_LEU_-rox1-down fragment was got and transformed into strain A10 by chemical transformation (Chen et al., 1992). The transformants were incubated on leucine-deficient synthetic agar plate at 30°C for screening. Then the transformants were selected randomly and purified by streaking on leucine-deficient screening plates. The verified transformant was called A10-rox1-NtCCD1-3. Strain A10-rox1-PhCCD1 was constructed using the same method as A10-rox1-NtCCD1-3. All the amplified genes, constructed plasmids and transformed strains used in this study were verified by sequencing. All primers used in this study are shown in Supplementary Table 1.

### NtCCD1-3 integration in the delta repeated sequences of the yeast genome

To construct the engineered yeast strain A10-*δ*-NtCCD1-3 with *NtCCD1-3* integration in the delta locus, the NtCCD1-3 cassette containing promoter *ADH1*, *NtCCD1-3* gene, and terminator *CYC1*, was first amplified from plasmid tpLADH1-NtCCD1-3 with primers NtCCD1-3-*δ*-F and NtCCD1-3-*δ*-R. Then, the yeast selection marker LEU2 cassette was amplified from the tpLADH1 plasmid by using LEU-*δ*-F and LEU-*δ*-R primers, and the δ-up and *δ*-down fragments were amplified from *S. cerevisiae* BY4741 genome using primers *δ*up-F/*δ*up-R and *δ*down-F/*δ*down-R, respectively. The above four cassettes and the *Eco*R I linearized pColdTF vector were infused by pEASY®-Basic Seamless Cloning and Assembly Kit. The transformants were screened on LB solid medium with 50 μg/ml ampicillin. The recombinant plasmid pColdTF-*δ*-NtCCD1-3-LEU2 was confirmed by *Eco*R I digestion and sequencing. After digestion with *Pst* I and *Bam*H I, the *δ*-up-P_ADH1_-NtCCD1-3-T_CYC1_-P_LEU_-LEU2-T_LEU_-*δ*-down fragment was purified and transformed into strain A10 by chemical transformation (Chen et al., 1992). Finally, the transformants were screened by incubating on leucine-deficient synthetic agar plate at 30°C, and confirmed by sequencing. The correct transformant was named A10-*δ*-NtCCD1-3, and used for the subsequent experiments.

### Predicted protein structure of NtCCD1-3 and sequence alignment

Using the amino acid sequence of NtCCD1-3 as the query, 46 templates for constructing the three-dimensional model of NtCCD1-3 were retrieved on the SWISS-MODEL server.^1^ The 9-cis-epoxycarotenoid dioxygenase viviparous 14 (VP14, PDB 3NPE) from *Zea mays*, which shared the highest sequence identity with NtCCD1-3, was selected as the modeling template. The homology model of NtCCD1-3 structure was constructed using the SWISS-MODEL server (Waterhouse et al., 2018). The NtCCD1-3 model showing highest global model quality estimation (GMQE) and lowest root mean square deviation (RMSD) against the VP14 structure was selected for the following experiments.

Sequence alignment was prepared based on the amino acid sequences of NtCCD1-3, PhCCD1, NtCCD1-2, OfCCD1, RdCCD1, AtCCD1, VvCCD1, ZmCCD1, VP14, CsCCD4 and CmCCD4 using Espript 3.0 (Robert and Gouet, 2014), and the secondary structure depiction was based on the model of NtCCD1-3 constructed above.

### Membrane interaction analysis

The three-dimensional structure model of NtCCD1-3 was compared with the crystal structure of VP14. Structural models of ten NtCCD1-3 mutations were constructed in the same way as the NtCCD1-3 model. Positioning of Proteins in Membranes Server was used to analyze the interaction between mutation proteins and membranes (Lomize et al., 2012), and wild-type NtCCD1-3 (WT) was used as control. The analysis parameters include membrane binding affinity (ΔG_transfer_), membrane penetration depth and tilt angle. ΔG_transfer_ is the free energy of insertion of the protein into the membrane from an aqueous solution (Messing et al., 2010). Tilt angle is the angle that a protein is relative to the cell membrane (Lomize et al., 2012).

### Site-directed mutagenesis

The mutant gene is divided into two parts from the mutation site and amplified separately, and then the two parts are fused by overlapping. Using tpLADH1-NtCCD1-3 plasmid as template, the first half of the *NtCCD1-3* mutated gene *K25A* was amplified with primers N3-F and K25A-R1, and the second half of that was amplified with primers K25A-F2 and N3-R. The PCR program was as follows: initial denaturation at 95°C for 2 min; followed by 35 cycles of denaturation at 94°C for 50s, annealing at 55–60°C for 30 s, and extending at 72°C for 2 min; and with a final extension for 10 min at 72°C. PCR products were purified with TIANgel Midi Purification Kit and assembled by overlap PCR (Gibson, 2011). The overlap PCR reaction system was as follows: each tube contained 50 μl reaction liquid composed of 2 μl first half *K25A* gene solution, 2 μl second half *K25A* gene solution, 1 μl N3-F and N3-R primers each, 1 μl Pfu DNA Polymerase, 5 μl buffer, 4 μl dNTPs, and 34 μl ddH_2_O. The PCR program was that initial denaturation at 95°C for 2 min, followed by 35 cycles of 94°C for 50s, 55–60°C for 30 s, 72°C for 2 min, and finally extension for 10 min at 72°C. The PCR product was recovered from 1% agarose gel, and ligated into the p*EASY*®-T1 cloning vector to obtain the vector T1-K25A. Vectors T1-K31A, T1-K38A, T1-K42A, T1-S128A, T1-K140M, T1-T155M, T1-Y157A, T1-Y157L, and T1-K164L for other mutant genes of *NtCCD1-3* were constructed in the same method.

Using each vector above as a template, primers N3mut-F and N3mut-R were used to amplify the ten mutated genes with *Nco* I and *Spe* I restriction sites, respectively. The PCR products were digested with *Nco* I and *Spe* I, and the vector pColdTF-rox1-NtCCD1-3-LEU2 was also digested with *Nco* I and *Spe* I. Then, the linearized vector backbone of pColdTF-rox1-NtCCD1-3-LEU2 without *NtCCD1-3* gene was recovered and ligated with the ten mutated gene fragments respectively, and transformed into TransT1 competent cells. The transformants were screened on solid LB medium with 50 μg/ml ampicillin and the plasmids was extracted and verified by *Nco* I and *Spe* I digestion. After sequencing, the confirmed recombinant vectors containing mutated gene sequences above were constructed, respectively. These recombinant vectors were digested with *Pst* I and *Sma* I to obtain ten rox1-up-mutation-LEU2-rox1-down fragments, and then the ten fragments were transformed the strain A10 to construct A10-rox1-K25A, A10-rox1-K31A, A10-rox1-K38A, A10-rox1-K42A, A10-rox1-S128A, A10-rox1-K140M, A10-rox1-T155M, A10-rox1-Y157A, A10-rox1-Y157L and A10-rox1-K164L.

### Pos5 and Uli1 expression

*Pos5* gene was codon-optimized and synthesized by GenScript (Nanjing, China) and then cloned into the vector tpLADH1 to acquire tpLADH1-Pos5. The P_TEF1_-Pos5-T_ADH1_ and P_GAP_-Uli1-T_CYC1_ cassette were amplified from tpLADH1-Pos5 and pUC57-Uli1 by using primers Pos5-F and Pos5-R, Uli1-F and Uli1-R, respectively. The pColdTF-rox1-NtCCD1-3-LEU2 was linearized by *Bgl* II, and fused with P_TEF1_-Pos5-T_ADH1_ and P_GAP_-Uli1-T_CYC1_ by homology arms, respectively. In brief, linearized pColdTF-rox1-NtCCD1-3-LEU2 was mixed with P_TEF1_-Pos5-T_ADH1_ cassette or P_GAP_-Uli1-T_CYC1_ cassette according to the instructions, and transformed into TransT1 competent cells after incubation at 25°C for 15 min according to protocols of pEASY^®^-Basic Seamless Cloning and Assembly Kit. The plasmids pColdTF-Pos5-rox1 and pColdTF-Uli1-rox1 were obtained and confirmed by sequencing. The correct transformants were stored at-80°C for subsequent experiments.

For the construction of strain A10-rox1-NtCCD1-3-POS5, the pColdTF-Pos5-rox1 plasmid was digested with *Pst* I and *Bam*H I to recover the integration rox1up-NtCCD1-3-LEU2-Pos5-rox1down fragment (7.6 Kb), then the fragment was transformed into *S. cerevisiae* A10. Similarly, the pColdTF-Uli1-rox1 plasmid was digested with *Bsm* I and *Sac* I to recover a 7.3 Kb fragment and transformed into *S. cerevisiae* A10 to obtain strain A10-rox1-NtCCD1-3-Uli1.

### Real-time quantitative PCR analysis of the *NtCCD1-3* gene

Strains A10-rox1-NtCCD1-3 and 9# A10-*δ*-NtCCD1-3 cells pellets in logarithmic growth phase were collected and used for real-time quantitative PCR (qPCR) assay. Total RNA extraction, cDNA synthesis, and qPCR were performed according to E.Z.N.A.^®^ Plant RNA Kit, PrimeScript™ RT reagent Kit, 2 × TSINGKE^®^ Master qPCR Mix (SYBR Green I) Kit, respectively. Primers NtCCD1-3-qPCR-F and NtCCD1-3-qPCR-R were used for NtCCD1-3 amplified, and Act1-qPCR-F and Act1-qPCR-R for the actin gene (*act1*) amplified. Differences in the expression of the *NtCCD1-3* and *act1* genes were calculated according to the 2^−ΔΔCT^ method (Livak and Schmittgen, 2001) using the *act1* gene as the reference.

### Fermentation and volatile apocarotenoids extraction

Strains A10, A10-rox1-NtCCD1-3, A10-rox1-PhCCD1, A10-*δ*-NtCCD1-3, A10-rox1-NtCCD1-3mut, A10-rox1-NtCCD1-3-Pos5, and A10-rox1-NtCCD1-3-Uli1 were inoculated into 20 ml yeast extract peptone dextrose (YPD) medium, respectively, and incubated at 28°C with shaking at 200 rpm for 48 h. After that, 0.4 ml culture above and 1 ml dodecane were transferred to 20 ml fresh YPD medium respectively, and finally incubated for 9 days at 28°C with shaking at 200 rpm. After fermentation, the organic phase was collected by centrifuging at 21,734 × g for 10 min and dehydrated by adding anhydrous sodium sulfate before gas chromatography–mass spectrometry (GC–MS) detection. The cells were collected and lyophilized for dry weight measurement. Three biological replicates were carried out for each sample.

For headspace solid phase micro-extraction gas chromatography–mass spectrometry (SPME-GC–MS) analysis of the volatile apocarotenoids, strain A10-*δ*-NtCCD1-3 was cultured on YPD solid medium at 28°C for 5 days, and 0.15 g cell pellet was collected for the analysis. Strain A10 was treated in the same way and used as the control.

### GC–MS analysis for ***β***-ionone quantification

The *β*-ionone produced by different strains was quantified by using GC–MS (Agilent 5977B, United States) equipped with DB-5MS column (Agilent, United States, 30 m × 0.25 mm ID, 0.25 μm film thickness). Helium was introduced as carrier gas with flow rate of 2 ml/min. The temperature program started at 100°C and held for 2 min, and then increased to 210°C at a rate of 10°C/min, up to 290°C at a rate of 20°C/min, and finally held for 5 min. The electron ionization energy was 70 eV. The mass range was set from m/z 40–400 with the ion source temperature 200°C. The *β*-ionone quantification was carried out by external standard method. The steps were as follows: standard curve of *β*-ionone dissolved in dodecane was prepared according to its peak areas of different concentrations; *β*-ionone in the samples of dodecane extraction liquid was detected by GC–MS, and the contents of *β*-ionone in samples were calculated on the basis of the standard curve above; finally, the titers and yields of *β*-ionone were calculated according to the volume of dodecane added for *β*-ionone extraction and the dry cell weight (DCW).

### SPME-GC–MS analysis of volatile apocarotenoids

The volatile apocaroteniods produced by A10-*δ*-NtCCD1-3 and the control A10 were analyzed by SPME-GC–MS. The SPME fibre, equipped with 65 μm polydimethylsiloxane/divinylbenzene (Supelco, United States), was put into a headspace vial with 0.15 g cell pellet. Then the vial was stirred with 250 rpm at 80°C for 20 min. The apocaroteniods in the fibre were detected by GC–MS (Agilent 5977B, United States) equipped with DB-5MS column (Agilent, United States, 30 m × 0.25 mm ID, 0.25 μm film thickness) using helium as carrier gas at a flow rate of 2 ml/min. The temperature program was as follows: 40°C held for 1.0 min, up to 250°C at a rate of 2°C/min, and held for 10.0 min. The electron ionization energy was 70 eV. The mass range was recorded from m/z 30–500, and the ion source temperature was 200°C. NIST 2.0 spectral library was used to retrieve the detected apocaroteniods, and *β*-ionone was identified by comparing mass spectrum and chromatographic mobility with that of the authentic standard.

## Results

### NtCCD1-3 from *N. tabacum* has high activity to cleave ***β***-carotene producing ***β***-ionone

**Figure 1:**
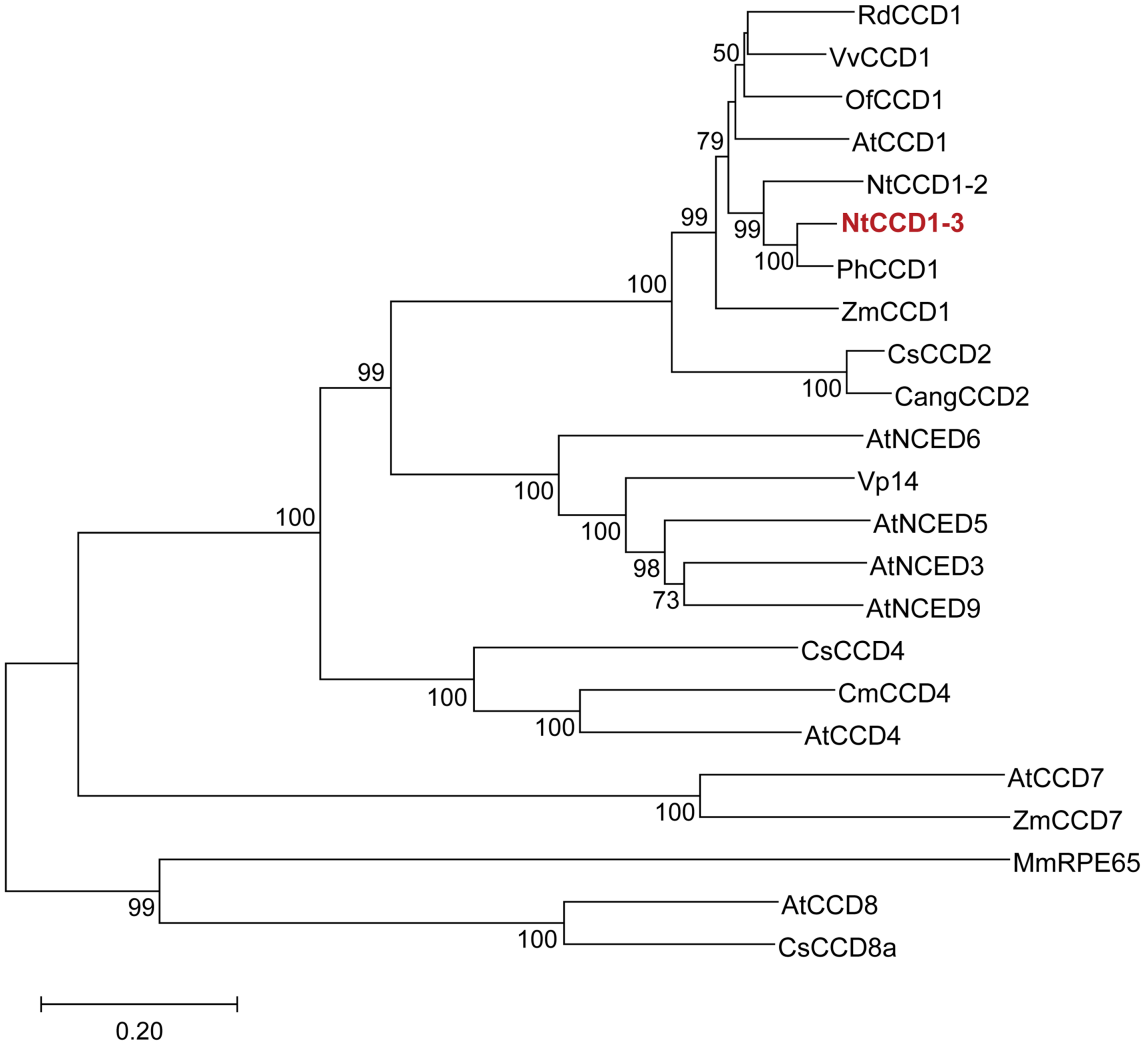
Phylogenetic analysis of CCDs from different organisms. NtCCD1-3 is in red and bold. The neighbor joining tree was generated by using MEGA X, based on 1,000 replicates. Bootstrap values above 50 are shown on the branches. The tree scale bar indicates the number of substitutions per site. Rd, *Rosa damascena*; Vv, *Vitis vinifera*; Of, *Osmanthus fragrans*; At, *Arabidopsis thaliana*; Nt, *Nicotiana tabacum*; Ph, *Petunia hybrida*; Zm, *Zea mays*; Cs, *Crocus sativus*; Cang, *Crocus angustifolius*; VP14, Viviparous 14; Cm, *Cucumis melo*; Mm, *Mus musculus*; RPE65, Retinal pigment epithelial protein 65 kDa.

Enzymes with close phylogenetic relationship may have similar catalytic activity to each other. Therefore, a phylogenetic tree of CCDs was constructed to screen the CCD1 which was closely related to PhCCD1 (Figure 1). The neighbor-joining phylogenetic tree showed that NtCCD1-3 from *N. tabacum* belonged to CCD1 clade, and clustered together with PhCCD1 with a high bootstrap value (100%). Therefore, we speculated that NtCCD1-3 might have similar catalytic properties to PhCCD1 and could be used for protein engineering study.

**Figure 2:**
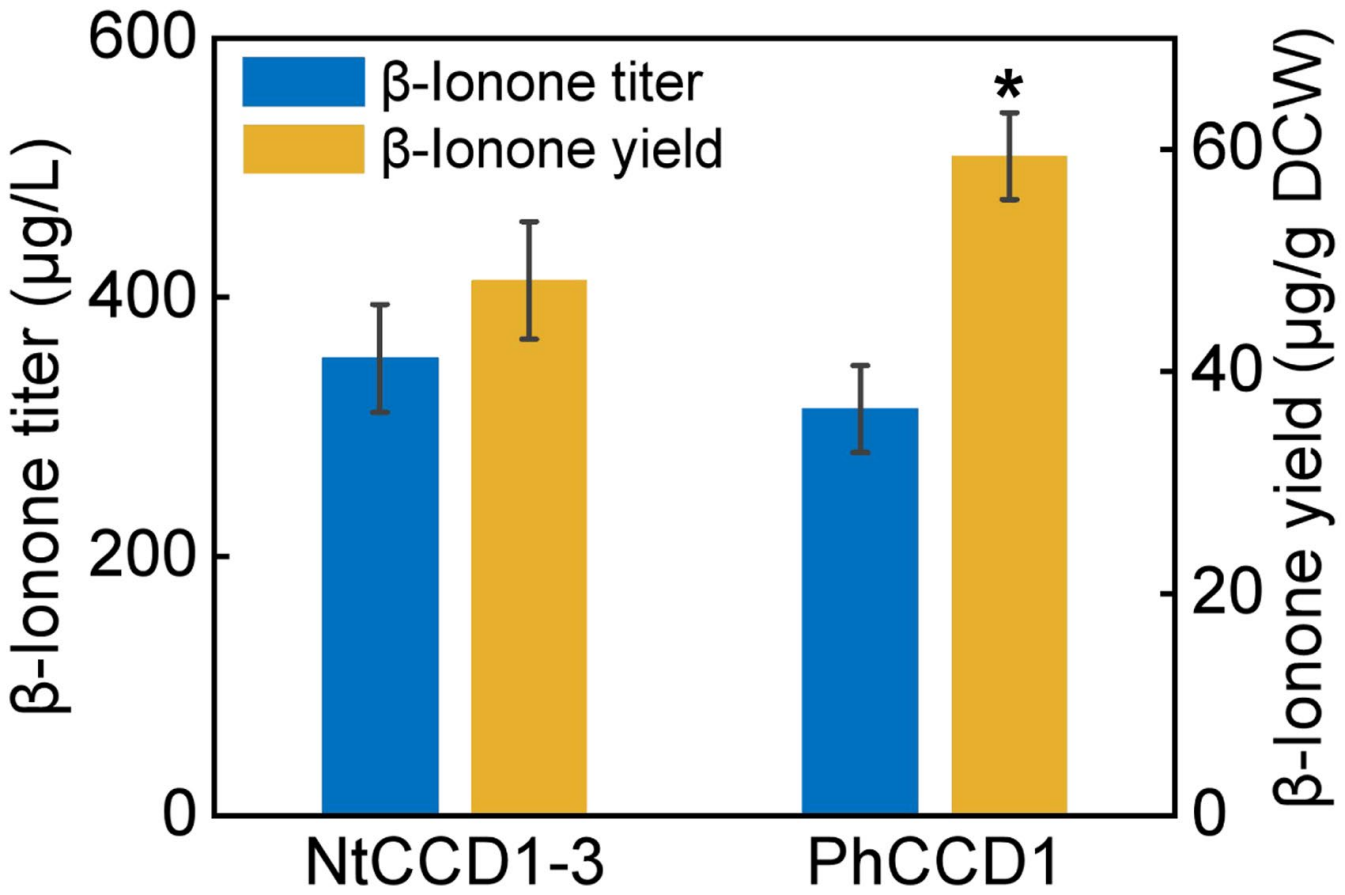
Comparison of *β*-ionone titer and yield of strains A10-rox1-NtCCD1-3 (NtCCD1-3) and A10-rox1-PhCCD1 (PhCCD1). Error bars represent the standard deviation of three biological replicates. *p* values were determined by one-way ANOVA (^*^p < 0.05).

To compare the NtCCD1-3 and PhCCD1 activity of cleaving carotenoid to produce *β*-ionone, strains A10-rox1-NtCCD1-3 and A10-rox1-PhCCD1 were constructed as mentioned above. The *β*-ionone titer of A10-rox1-NtCCD1-3 was 352.8 ± 41.7 μg/l, which increased 12.4% comparing with that of A10-rox1-PhCCD1 (313.8 ± 33.6 μg/l) (Figure 2). While, the *β*-ionone yield of A10-rox1-NtCCD1-3 was lower than that of A10-rox1-PhCCD1. These results indicate that the catalytic activity of NtCCD1-3 is similar to that of PhCCD1. As a result, NtCCD1-3 was chosen for the candidate to synthesize *β*-ionone.

### The delta integrating site for NtCCD1-3 gene is suitable for enhancing the yield of ***β***-ionone

**Figure 3:**
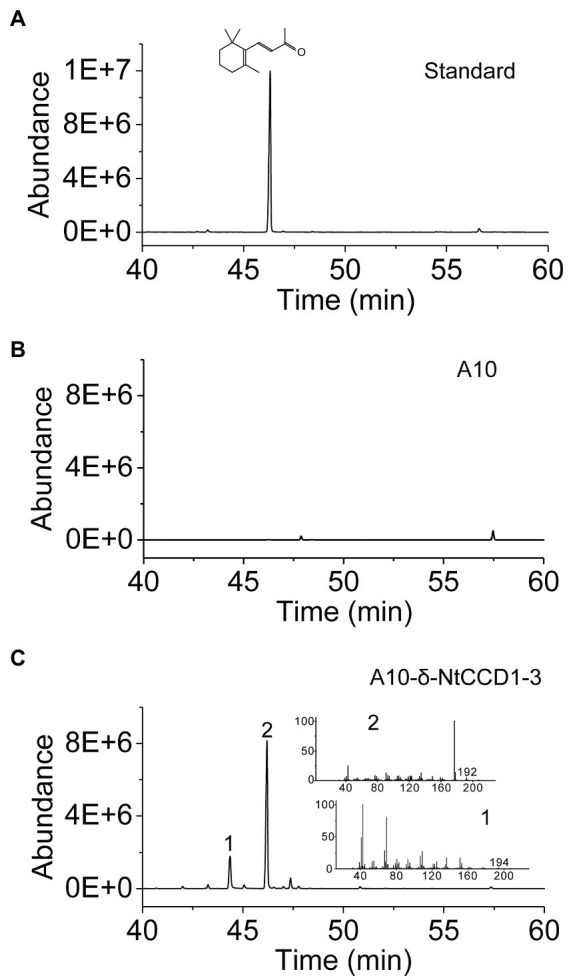
SPME-GC–MS analysis of authentic standard *β*-ionone **(A)** and volatile products in the strain A10 **(B)** as well as the 9# recombinant strain of A10-*δ*-NtCCD1-3 **(C)**. The compound 1 has an identical mass spectrum to that of the geranylacetone, and compound 2 has an identical mass spectrum and a chromatographic mobility identical to that of the authentic standard *β*-ionone.

Integrating *NtCCD1-3* gene into the multi-copy delta site may promote the yield of *β*-ionone. Therefore, we integrated *NtCCD1-3* at the delta site of the yeast genome to construct strain A10-*δ*-NtCCD1-3. The A10-*δ*-NtCCD1-3 producing the highest yield of *β*-ionone was screened by SPME-GC–MS (Supplementary Figure 2). The 9# transformant of A10-*δ*-NtCCD1-3 producing the most *β*-ionone whose peak area was 4.89 × 10^8^, while the control strain A10 produced little *β*-ionone (Figure 3). In the same time, geranylacetone was also detected in the A10-*δ*-NtCCD1-3 (Figure 3C), but not in the A10 strain (Figure 3B). In addition, pseudoionone and 6-methyl-5-heptene-2-one (MHO) were also detected in the A10-*δ*-NtCCD1-3 (data not shown).

**Figure 4:**
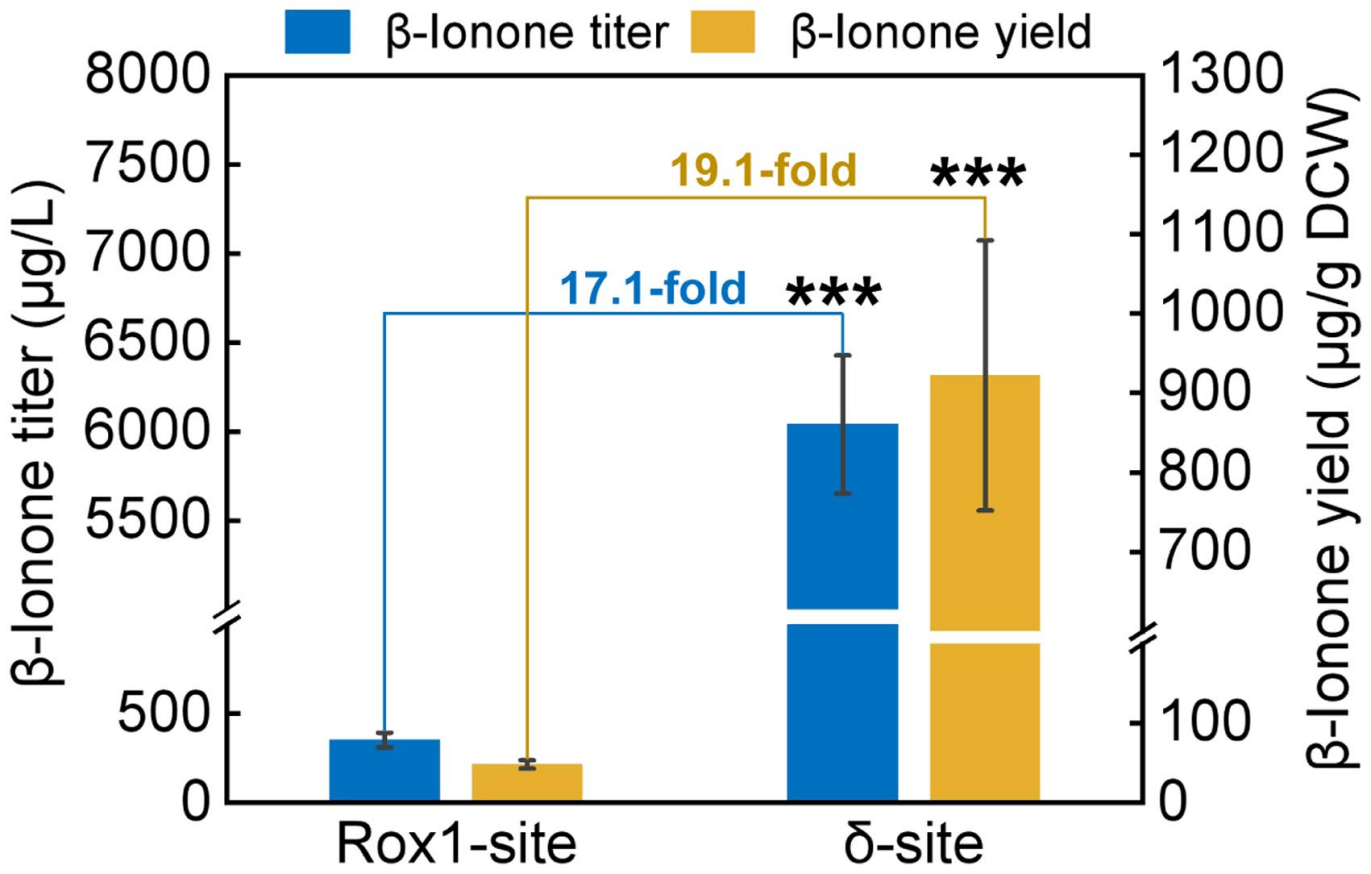
The production of *β*-ionone by the strains A10-rox1-NtCCD1-3 (Rox1-site) and 9# A10-*δ*-NtCCD1-3 (*δ*-site). Error bars represent the standard deviation of three biological replicates. *p* values were determined by one-way ANOVA (^***^p < 0.001).

To investigate the impact of integration site on *β*-ionone synthesis, the *β*-ionone production by A10-rox1-NtCCD1-3 and A10-*δ*-NtCCD1-3 was quantified by GC–MS (Figure 4). Both titer and yield of *β*-ionone in A10-*δ*-NtCCD1-3 were higher than that in A10-rox1-NtCCD1-3. The titer of *β*-ionone reached to 6.0 mg/ml in A10-*δ*-NtCCD1-3, which was 17.1-fold of that in A10-rox1-NtCCD1-3. Meanwhile, the yield of *β*-ionone was 0.9 mg/g DCW in A10-*δ*-NtCCD1-3, which was 19.1-fold of that in A10-rox1-NtCCD1-3. These results suggest that the insertion site delta for NtCCD1-3 gene is more suitable for enhancing the yield of *β*-ionone compared to the integrating site rox1.

### Mutants in α1 and α5 membrane-bonding domains promote ***β***-ionone production

**Figure 5:**
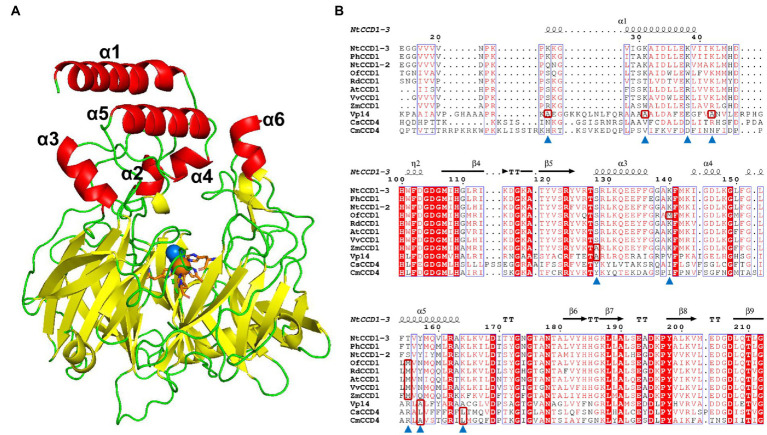
The simulated structure of NtCCD1-3 and its sequence alignment with other CCDs from different plants. **(A)** Structure of NtCCD1-3 simulated by SWISS-MODEL server. The Fe^2+^ cofactor is shown as orange sphere, and the dioxygen binding with Fe^2+^ are shown as blue spheres. The four conserved histidine residues binding to Fe^2+^ are marked with orange sticks. **(B)** Amino acid sequence alignment of NtCCD1-3 with other plant-derived CCDs. The amino acid residues selected for site-directed mutagenesis are marked with blue triangles. The hydrophobic amino acids which the selected amino acid residues were mutated into are shown in the red boxes.

The simulated model of NtCCD1-3 with highest GMQE, showing the lowest RMSD against the VP14 structure was selected for membrane-binding domains confirmation and sequence alignment. Like other CCDs structures, the NtCCD1-3 also has a seven bladed *β*-propeller structure with Fe^2+^ ion in its center as the cofactor, which is coordinated bonding with four conserved histidine residues, forming its active site (Figure 5A). The membrane-binding domains of NtCCD1-3 are two antiparallel *α*-helix *α*1 and *α*5, corresponding to helices *α*1 and *α*3 of VP14 structure (Figure 5A). Helices *α*1 and *α*5 of NtCCD1-3 are formed by amino acid residues 24–40 and 151–164, respectively. Besides *α*1 and *α*5, other four helices *α*2, *α*3, *α*4 and *α*6 are also involved in forming the cape of NtCCD1-3 (Figure 5A).

To improve the membrane binding ability of NtCCD1-3, four hydrophilic amino acid residues Lys-25, Lys-31, Lys-38 and Lys-42 in the *α*1 helix, three hydrophilic amino acid residues Thr-155, Tyr-157 and Lys-164 in the α5 helix, as well as hydrophilic amino acid residues Ser-128 in the *α*3 helix and Lys-140 in the loop structure between α3 and α4 helices were selected for site-directed mutagenesis. Aside from Lys-38 mutated into alanine, the other residues above were mutated into the hydrophobic amino acids at the corresponding positions of other CCDs (Figure 5B shown in the red boxes). Finally, mutations K25A, K31A, K38A, K42A, S128A, K140M, T155M, Y157A, Y157L and K164L were acquired.

**Figure 6:**
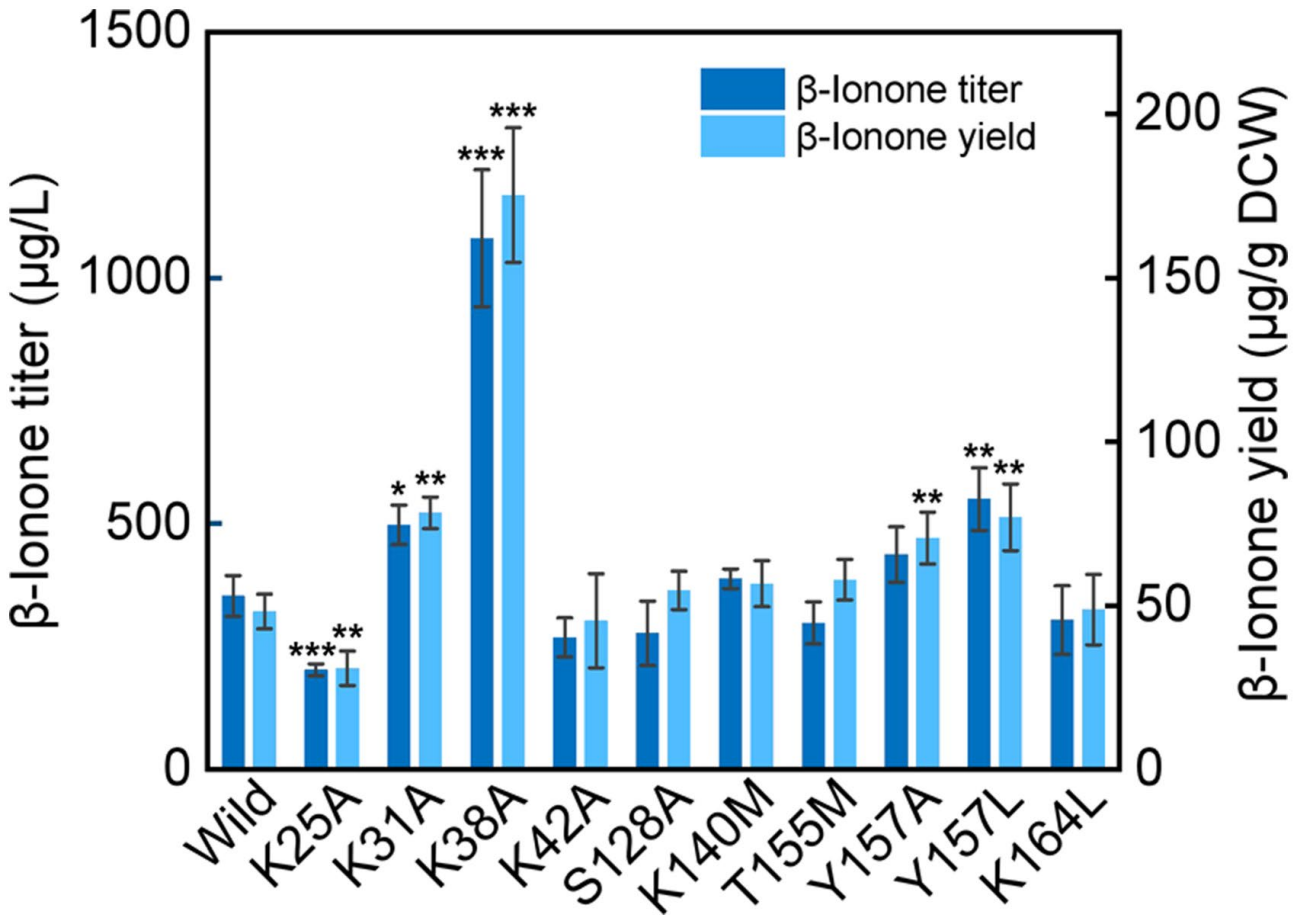
*β*-Ionone production in the wild-type strain of NtCCD1-3 (Wild) and the mutants. Error bars represent the standard deviation of three biological replicates. *p* values were determined by one-way ANOVA (^*^p < 0.05; ^**^p < 0.01; ^***^p < 0.001).

Compared with the wild type NtCCD1-3 (“Wild” in short), four mutants K31A, K38A, Y157A and Y157L showed significant promotion of *β*-ionone production (*p* < 0.05; Figure 6). Mutant K38A showed the highest *β*-ionone production ability, and the titer and yield were 1.08 mg/l and 0.18 mg/g DCW, respectively, which were 3.1 and 3.6 times higher than that of the Wild. The *β*-ionone titer and yield of mutant Y157L were 0.55 mg/l and 0.08 mg/g DCW, which were 1.6 times higher than those of the Wild NtCCD1-3. K31A mutant increased the titer and yield of *β*-ionone by 1.4 and 1.6-fold, respectively. The reason might be that the membrane penetration depth of K38A (4.3 ± 1.3 Å) is bigger than that of the wild protein (4.1 ± 1.2 Å; Table 2). The results of K31A and Y157A also support this speculation (Table 2). The membrane penetration depth of K25A (3.5 ± 1.6 Å) is smaller than that of the wild protein (4.1 ± 1.2 Å), and that may be the reason why *β*-ionone titer and yield of mutant K25A decreased significantly (*p* < 0.01) compared to the Wild. These results suggest that Lys-38, Tyr-157 and Lys31 may be the key amino acid residues that affect the activity of NtCCD1-3 by influencing its binding to cell membrane. And three of the amino acid residues Lys-25, Lys-31 and Lys-38 locate in helix *α*1 of NtCCD1-3, and Tyr-157 in helix *α*5, which confirms helices *α*1 and *α*5 involving in protein penetrating into the cell membrane. The results also imply that helix *α*1 may play key role in the catalytic process. These results are consistent with VP14, whose helix *α*1 deletion or disruption resulted in loss of ABA biosynthesis (Messing et al., 2010). Unlike PhCCD1, the K164L mutant of NtCCD1-3 did not present significant increase of *β*-ionone production (Werner et al., 2019). The reason remains unknown.

**Table 2:** Silico analysis the membrane interaction parameters of NtCCD1-3 and its mutants.

NtCCD1-3	ΔG_transfer_ (kcal/mol)	Membrane penetration depth (Å)	Tilt angle (^o^)
Wild	−6.7	4.1 ± 1.2	30 ± 3
K25A	−8.3	3.5 ± 1.6	19 ± 7
K31A	−10.4	4.3 ± 1.2	26 ± 7
K38A	−6.6	4.3 ± 1.3	30 ± 2
K42A	−7.3	3.0 ± 0.7	32 ± 3
S128A	−6.7	4.1 ± 1.2	30 ± 3
K140M	−6.5	4.4 ± 1.6	30 ± 2
T155M	−6.7	4.1 ± 1.1	30 ± 3
Y157A	−6.4	4.2 ± 1.2	31 ± 3
Y157L	−5.9	3.6 ± 1.7	31 ± 7
K164L	−6.7	4.2 ± 0.8	30 ± 5

### Overexpression of Pos5 improves ****β****-ionone synthesis

Since the heterologous expression of many heterologous genes in engineered strains may cause the increase of unfolded or misfolded proteins. We speculate that overexpression Uli1 (a kind of unfolded protein response-inducible protein) might promote the *β*-ionone production by reducing the ratio of unfolded or misfolded proteins which are involving in *β*-ionone synthesis. Therefore, two strains A10-rox1-NtCCD1-3-Pos5 and A10-rox1-NtCCD1-3-Uli1 were constructed to investigate the influence of Pos5 and Uli1 overexpression on *β*-ionone synthesis. Compared with the control (A10-rox1-NtCCD1-3), the yield of *β*-ionone in A10-rox1-NtCCD1-3-Pos5 strain was significantly increased, 1.5 times than that of the control (Supplementary Figure 3). While, the titer and yield of *β*-ionone producing by A10-rox1-NtCCD1-3-Uli1 strain did not change significantly. These results suggest that Pos5 overexpression could increase the synthesis of *β*-ionone.

## Discussion

CCDs are key enzymes in biosynthesis of *β*-ionone, and their activity is still the main factor restricting the yield of *β*-ionone. However, there are few studies on the CCDs with high activity for *β*-ionone biosynthesis, and also few reports on improving the catalytic activity of CCDs by protein engineering technology. Therefore, this study focused on the yield improvement of *β*-ionone, and several strategies including exploring CCDs with high catalytic activity, integration site optimization, protein engineering of the NtCCD1-3 and NADPH supply, were carried out.

Studies have shown that PhCCD1 had the highest activity to cleave carotene producing *β*-ionone among PhCCD1, AtCCD1, VvCCD1, and OfCCD1 (Zhang et al., 2018). So researchers mostly chose PhCCD1 as a high-efficient enzyme to biosynthesize *β*-ionone (Ye et al., 2018; Zhang et al., 2018; Werner et al., 2019; Lu et al., 2020). In this study, NtCCD1-3 from *N. tabacum* was selected, and its amino acid sequence shares 93% identity with PhCCD1, which has high catalytic activity of cleaving *β*-carotene to produce *β*-ionone. The titer of *β*-ionone produced by NtCCD1-3 was even higher than that of PhCCD1. Like *P. hybrida*, *N. tabacum* also belongs to Solanaceae. These findings imply that the CCD1 proteins from Solanaceae plants may have higher activity to cut *β*-carotene at the 9,10 (9′,10′) position than these from other families.

In the A10-*δ*-NtCCD1-3 strain, *β*-ionone, geranylacetone, pseudoionone, as well as MHO were detected by SPME-GC–MS. Hence, we proposed that NtCCD1-3 may cleave *β*-carotene, phytoene as well as lycopene at the 9,10 (9′,10′) double bonds to generate *β*-ionone, geranylacetone, and pseudoionone respectively, and also may cleave lycopene at the 5,6 (5′,6′) positions to produce MHO (Supplementary Figure 4). These results are consistent with the apocarotenoids produced by ZmCCD1, AtCCD1, LeCCD1A, and LeCCD1B. And, it was reported that ZmCCD1 cleaved *β*-carotene, *δ*-carotene, lycopene as well as *ζ*-carotene at the 9,10 (9′,10′) double bonds, and also cleaved *δ*-carotene and lycopene at the 5,6 (5′,6′) double bonds (Vogel et al., 2008). In addition, CmCCD1 and RdCCD1 showed the cleavage of phytoene at the 9,10 (9′,10′) sites to generate geranylacetone (Ibdah et al., 2006; Huang et al., 2009).

Rox1 is a transcriptional factor that inhibits the genes of MVA pathway (Henry et al., 2002; Montañés et al., 2011), so the *rox1* gene position was selected as the NtCCD1-3 integration site to remove its inhibition to MVA pathway. Meanwhile, Gao et al., (2017) have reported that increasing the gene copy numbers of rate-limiting enzymes could enhance the yield of *β*-carotene. In this study, we found that the multiple integrating delta site of NtCCD1-3 was more beneficial to *β*-ionone production than the single integrating site rox1. The delta integrating site of NtCCD1-3 promotes the yield of *β*-ionone by 19.1-fold compared to the rox1 site, and the titer by 17.1-fold. The reason may be that the NtCCD1-3 gene copy numbers of A10-*δ*-NtCCD1-3 are more than that of A10-rox1-NtCCD1-3. Therefore, the relative expression level of NtCCD1-3 in these two strains were detected by qPCR, as expected, it was found that the expression level of NtCCD1-3 in strain A10-*δ*-NtCCD1-3 was significantly higher (*p* < 0.001) than that in A10-rox1-NtCCD1-3 (Supplementary Figure 5). This in turn proves that NtCCD1-3 catalytic step is the rate-limiting step in *β*-ionone biosynthesis pathway, which is consistent with the conclusion reported previously (Czajka et al., 2018).

In the *β*-ionone synthesis pathway constructed in *S. cerevisiae*, the step from hydroxymethylglutaryl-CoA to mevalonate is the key rate-limiting step (Kampranis and Makris, 2012), which requires NADPH as the cofactor. Pos5 a NADH kinase participates in the synthesis of NADPH (Outten and Culotta, 2003). It has been reported that overexpression of Pos5 can significantly increase the yield of *β*-carotene (Zhao et al., 2015). Since *β*-carotene is a precursor of *β*-ionone, we speculated that overexpression of Pos5 in *S. cerevisiae* may also increase the yield of *β*-ionone. In this work, we found that overexpression of the NADPH-responsible Pos5 could increase the yield of *β*-ionone by 1.5 times. The reason may be that the expression of Pos5 promotes the NADPH production, which in turn accelerates the catalytic reaction from hydroxymethylglutaryl-CoA to mevalonate. And this result was consistent with previous report that the yield of *β*-carotene was significantly increased when the *Pos5* gene was overexpressed in *S. cerevisiae* (Zhao et al., 2015). Uli1 is a kind of unfolded protein response-inducible protein that helps reduce misfolded or unfolded proteins (Ghaemmaghami et al., 2003). In the study, we found that overexpression of Uli1 could not increase the yield of *β*-ionone.

In summary, the NtCCD1-3 from *N. tabacum* possessing high ability of *β*-carotene cleavage was investigated. We found that NtCCD1-3 can cleave multiple caroteniods at the 9,10 (9′,10′) double bonds, and also had the ability of cleaving lycopene at the 5,6 (5′,6′) positions. To increase the production of *β*-ionone, several experiments were carried out. Finally, three main conclusions were drawn: (1) the delta integrating site for NtCCD1-3 gene was suitable for enhancing the yield of *β*-ionone, showing 19.1-fold increase compared with rox1 site; (2) mutants in membrane-bonding domains *α*1 and *α*5 of NtCCD1-3 promoted *β*-ionone production, and the K38A one showed over 3-fold increase than the strain with wild protein; (3) overexpression of Pos5 improved *β*-ionone yield up to 1.5 times. In the future, multiple sites mutants may be constructed to further increase *β*-ionone yield. And the strategy of integrating CCD1 in delta site and protein engineering combination can be a employed for *β*-ionone production. These results provide valuable evidence for biosynthesis of *β*-ionone and other apocarotenoids in microbial factories, and further increase the understanding of CCD1 proteins.

## Data availability statement

The datasets presented in this study can be found in online repositories. The names of the repository/repositories and accession number(s) can be found in the article/Supplementary material.

## Author contributions

MW and XH designed the experiments and edited the paper. XG wrote the paper and contributed to formal analysis. FL performed the experiments and wrote the paper. YL performed the protein structure analysis. All authors contributed to the article and approved the submitted version.

## Funding

This work was supported by the National Natural Science Foundation of China (31760189 and 32060531) and the Yunnan Fundamental Research Project (202101AT070279).

## Conflict of interest

XG was employed by company Tobacco Yunnan Industrial Co., Ltd.

The remaining authors declare that the research was conducted in the absence of any commercial or financial relationships that could be construed as a potential conflict of interest.

## Publisher’s note

All claims expressed in this article are solely those of the authors and do not necessarily represent those of their affiliated organizations, or those of the publisher, the editors and the reviewers. Any product that may be evaluated in this article, or claim that may be made by its manufacturer, is not guaranteed or endorsed by the publisher.

## Footnotes

^1^ https://swissmodel.expasy.org/
