# Supplementary material for: Characteristics of NtCCD1-3 from tobacco, and protein engineering of the CCD1 to enhance β-ionone production in yeast
Source: Front Microbiol. 2022 Sep 23;13:1011297. doi: 10.3389/fmicb.2022.1011297 (PMC9539813; doi:10.3389/fmicb.2022.1011297)
Supplement: Supplementary file 1 [file Data_Sheet_1.docx]

**
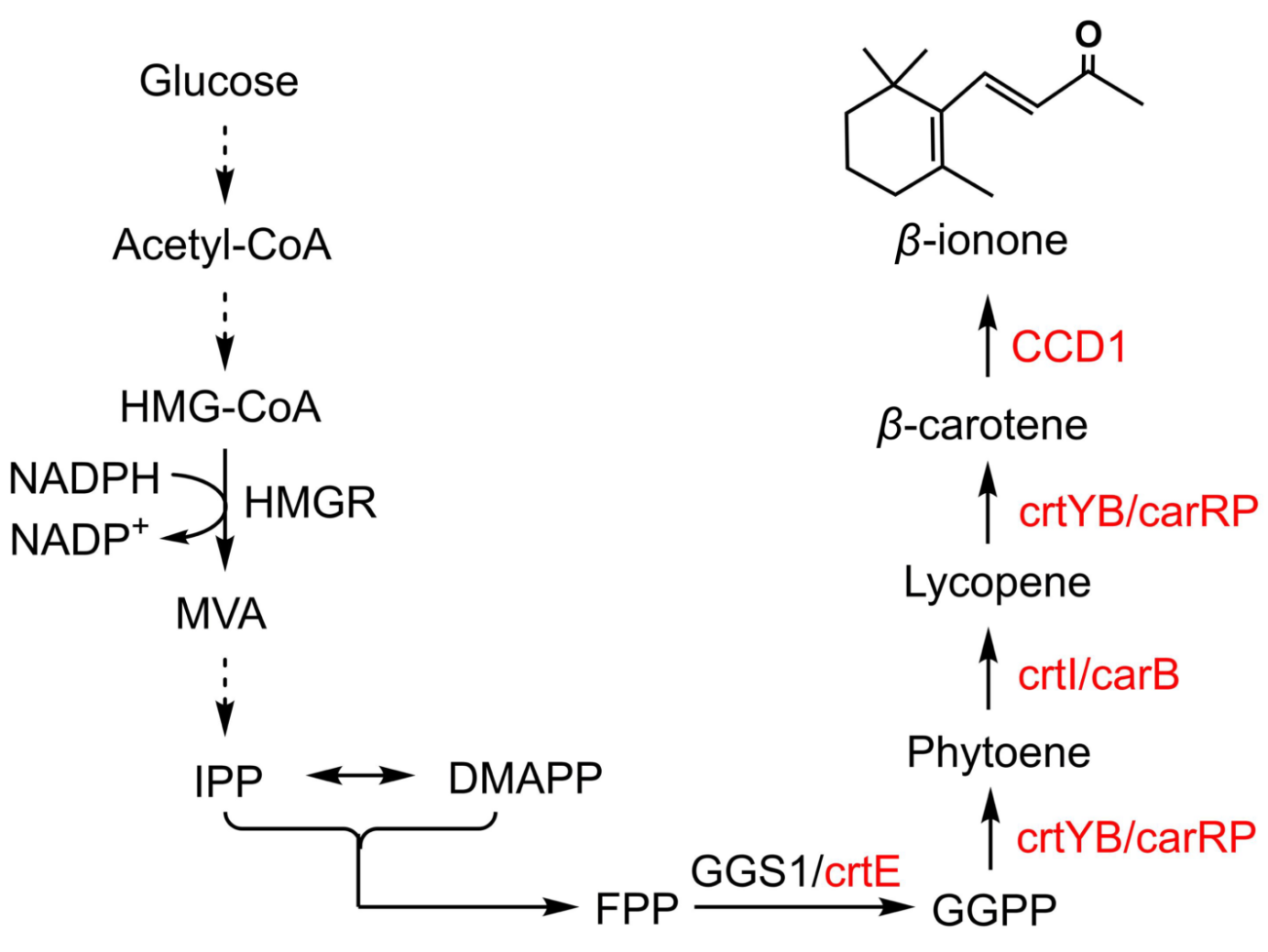
**

**Supplementary Figure 1**

**
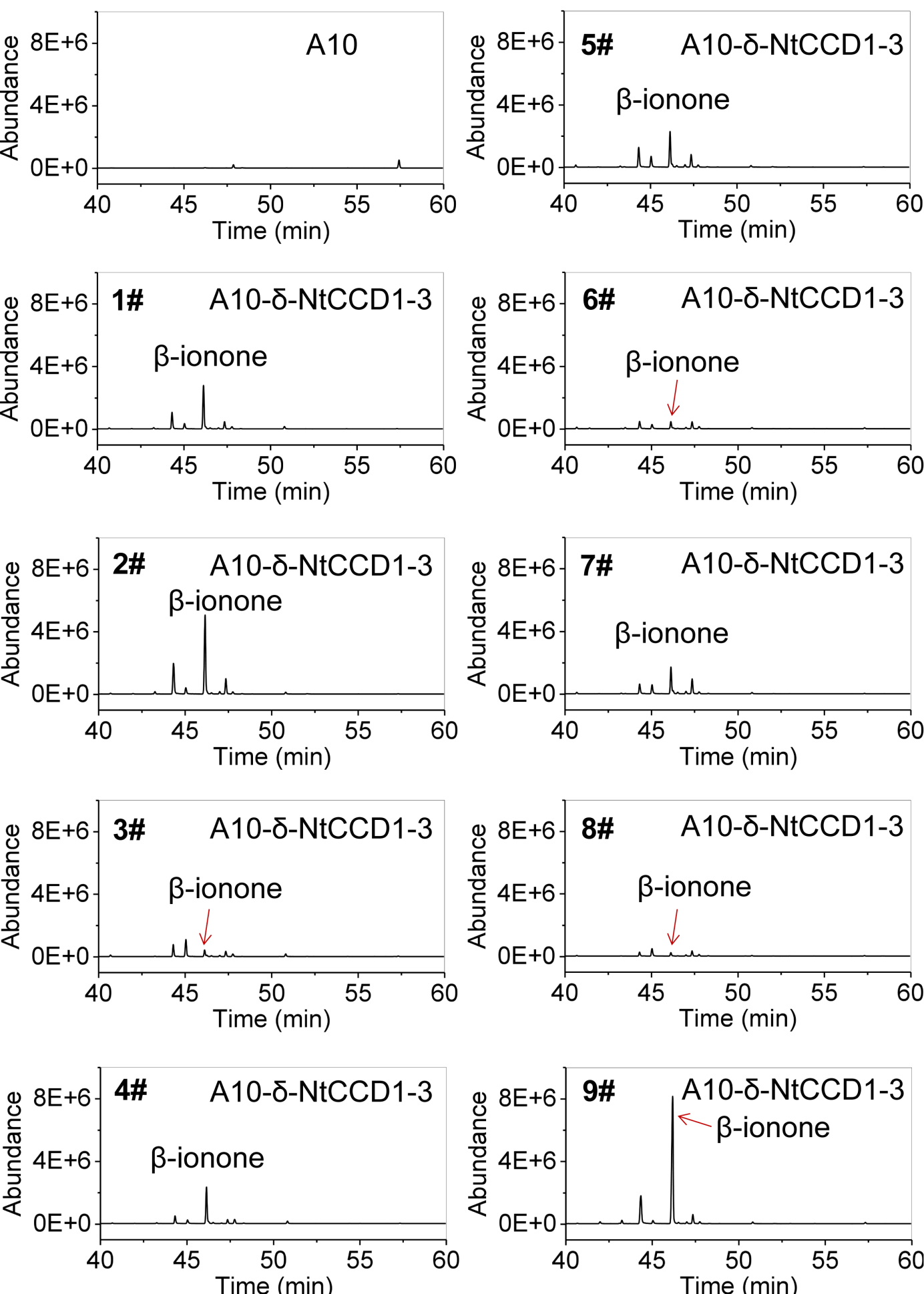
**

**Supplementary Figure 2**

**
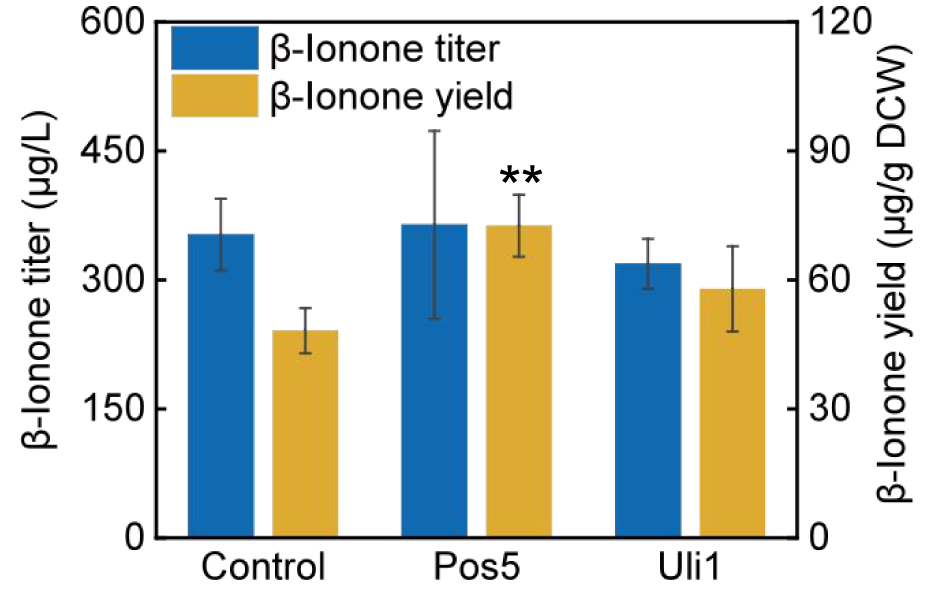
**

**Supplementary Figure 3**

**
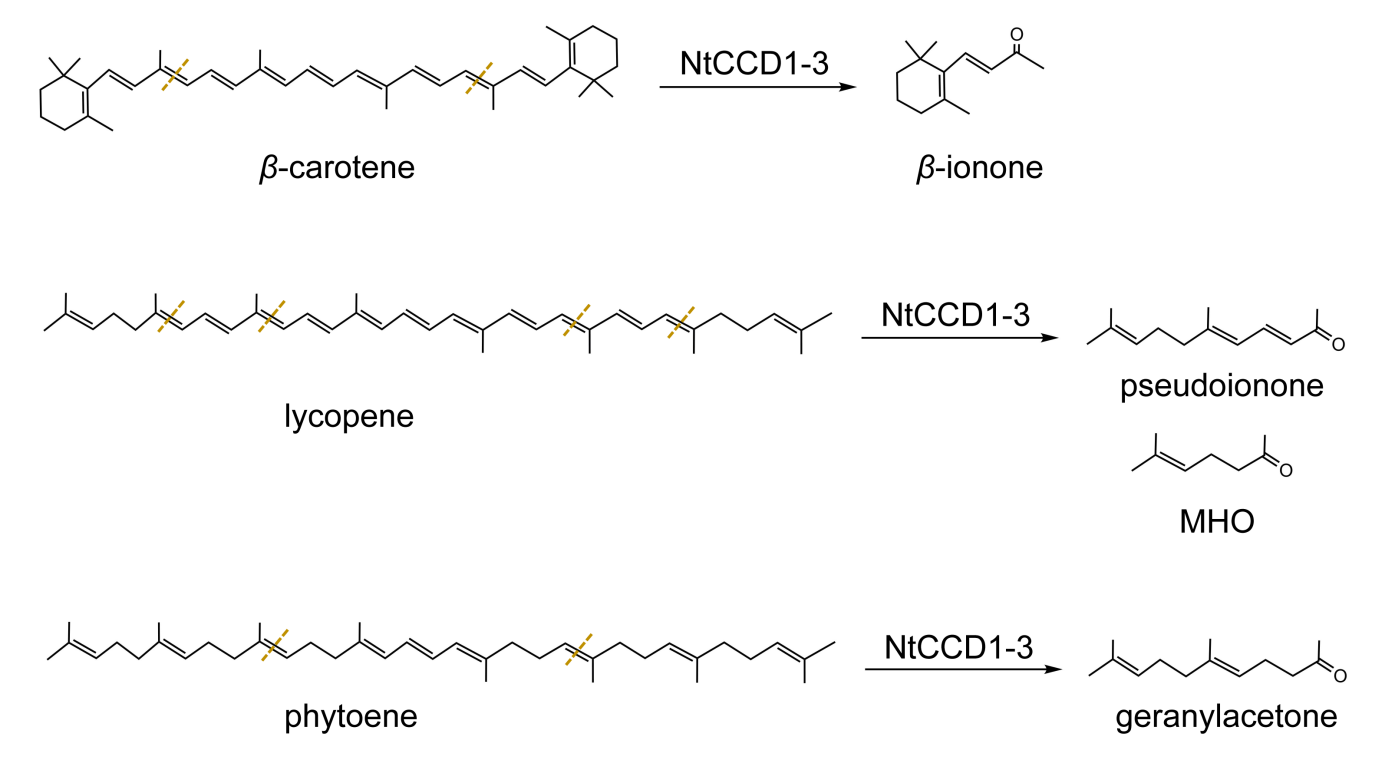
**

**Supplementary Figure 4**

**
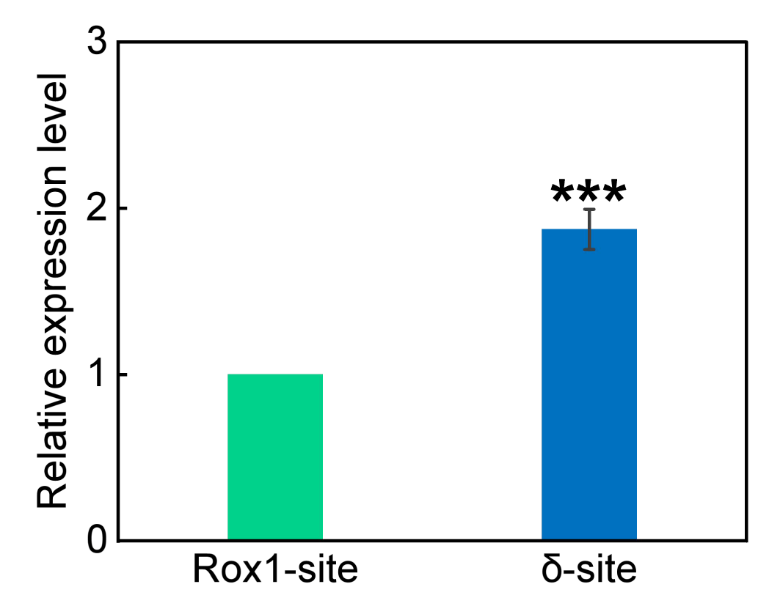
**

**Supplementary Figure 5**

**Supplementary Table 1 |** Primers used in this study.

| Primers | Sequence (5’ to 3’) |
| --- | --- |
| rox1-F | ATGAATCCTAAATCCTCTACACCTA |
| rox1-R | TCATTTCGGAGAAACTAGGCTAGTT |
| rox1up-F | cggtaccctcgagggatccgATGAATCCTAAATCCTCTACACCTAAG |
| rox1up-R | GTACACCCGGAAACAACAAAAGGATTTAACAGAAGGAATAGGCAACCTC |
| rox1down-F | GAAACGGCCTTAACGACGTAGTCGATTACTTCTAACTATATGGTCTCCAG |
| rox1down-R | CTAGACTGCAGGTCGACAAGCTTGTCATTTCGGAGAAACTAGGCTAG |
| NtCCD1-3-infusion-F | GAGGTTGCCTATTCCTTCTGTTAAATCCTTTTGTTGTTTCCGGGTGTAC |
| NtCCD1-3-infusion-R | CGATACCTGAGTATTCCCACAGTTGCAAATTAAAGCCTTCGAGCGTCCC |
| LEU-infusion-F | GGGACGCTCGAAGGCTTTAATTTGCAACTGTGGGAATACTCAGGTATCG |
| LEU-infusion-R | CTGGAGACCATATAGTTAGAAGTAATCGACTACGTCGTTAAGGCCGTTTC |
| PhCCD1-infusion-F | Same to NtCCD1-3-infusion-F |
| PhCCD1-infusion-R | Same to NtCCD1-3-infusion-R |
| *δ*up-F | tcggtaccctcgagggatccgaattTGTTGGAATAGAAATCAACTATCATCTACTAAC |
| *δ*up-R | ACAACAAAAGGATAGGATATAGGAATCCTCAAAATGGAATCTATATTTCTACATAC |
| *δ*down-F | GACGTAGTCGATTTCTGTATACCTAATATTATAGCCTTTATCAACAATGGAATCC |
| *δ*down-R | gactgcaggtcgacaagcttgaattATGGGGGTTCTCTGGAACAGC |
| NtCCD1-3-*δ*-F | CCATTTTGAGGATTCCTATATCCTATCCTTTTGTTGTTTCCGGGTGTAC |
| NtCCD1-3-*δ*-R | CGATACCTGAGTATTCCCACAGTTGGATCCGCAAATTAAAGCCTTCGAG |
| Leu-*δ*-F | CTCGAAGGCTTTAATTTGCGGATCCAACTGTGGGAATACTCAGGTATCG |
| Leu-*δ*-R | GGCTATAATATTAGGTATACAGAAATCGACTACGTCGTTAAGGCCG |
| N3-F | ATGGGTAGAAAGGAAGAAGATGATACTGTAGAAAG |
| N3-R | TCATAACTTGGCTTGTTCTTGGATTTGTTCTTCGGTG |
| K25A-R1 | CAATTGCTTTACCTATTACACCTTTAGCAGGCTTTGGATTGACTA |
| K25A-F2 | TAGTCAATCCAAAGCCTGCTAAAGGTGTAATAGGTAAAGCAATTG |
| K31A-R1 | TCTAACAAATCAATTGCTGCACCTATTACACCTTTCTTAG |

**Supplementary Table 1 |** *Continued.*

| Primers | Sequence (5’ to 3’) |
| --- | --- |
| K31A-F2 | CTAAGAAAGGTGTAATAGGTGCAGCAATTGATTTGTTAGA |
| K38A-R1 | CATGCATCAATTTAATAATAACTGCTTCTAACAAATCAATTGC |
| K38A-F2 | GCAATTGATTTGTTAGAAGCAGTTATTATTAAATTGATGCATG |
| K42A- R1 | GGTTTAGTAGAATCATGCATCAATGCAATAATAACTTTTTCTAAC |
| K42A- F2 | GTTAGAAAAAGTTATTATTGCATTGATGCATGATTCTACTAAACC |
| S128A-R1 | CCGAAAAATTCTTCTTGTTTCAATCTTGCAGTTCTAACGTATCTTGAAAC |
| S128A-F2 | GTTTCAAGATACGTTAGAACTGCAAGATTGAAACAAGAAGAATTTTTCGG |
| K140M-R1 | GTCACCAATTTTCATGAACATAGCACCACCGAAAAATTCTTC |
| K140M-F2 | GAAGAATTTTTCGGTGGTGCTATGTTCATGAAAATTGGTGAC |
| T155M-R1 | GCTCTCAAAACTTGCATATAAACCATAAACAAACCAAACAAACCTTTC |
| T155M-F2 | GAAAGGTTTGTTTGGTTTGTTTATGGTTTATATGCAAGTTTTGAGAGC |
| Y157A-R1 | CAATTTAGCTCTCAAAACTTGCATAGCAACAGTAAACAAACCAAACAAAC |
| Y157A-F2 | GTTTGTTTGGTTTGTTTACTGTTGCTATGCAAGTTTTGAGAGCTAAATTG |
| Y157L-R1 | CTTTCAATTTAGCTCTCAAAACTTGCATCAAAACAGTAAACAAACCAAAC |
| Y157L-F2 | GTTTGGTTTGTTTACTGTTTTGATGCAAGTTTTGAGAGCTAAATTGAAAG |
| K164L-R1 | CCATAAGTAATATCTAAAACTTTCAACAAAGCTCTCAAAACTTGCATAT |
| K164L-F2 | ATATGCAAGTTTTGAGAGCTTTGTTGAAAGTTTTAGATATTACTTATGG |
| N3mut-F | CATGCCATGGATGGGTAGAAA |
| N3mut-R | GGACTAGTTCATAACTTGGCTTGTT |
| Uli1-F | GATTACTTCTAACTATATGGTCTCCATCATTATCAATACTCGCCATTTCAAAG |
| Uli1-R | CATGCGTCAAAGGTAGTCCACTTAAAGCAAATTAAAGCCTTCGAGC |
| Pos5-F | GATTACTTCTAACTATATGGTCTCCACCACACACCATAGCTTCAAAATG |
| Pos5-R | GCGTCAAAGGTAGTCCACTTAAACCGGTAGAGGTGTGGTCAATAAGAG |
| NtCCD1-3-qPCR-F | GATGGTGACGGTATGATTCATGG |
| NtCCD1-3-qPCR-R | CCAAACAAACCTTTCAAGTCACC |
| Act1-qPCR-F | GAATTGAGAGTTGCCCCAGA |
| Act1-qPCR-F | GGCTTGGATGGAA ACGTAGA |
